# Prognostic indicators for feline craniofacial trauma: a retrospective study of 114 cases

**DOI:** 10.3389/fvets.2023.1190474

**Published:** 2023-05-12

**Authors:** Jennifer L. Kelley, Naomi K. Hoyer, Sangeeta Rao, Jennifer E. Rawlinson

**Affiliations:** Department of Clinical Sciences, Veterinary Dentistry and Oral Surgery, Colorado State University College of Veterinary Medicine and Biomedical Sciences, Fort Collins, CO, United States

**Keywords:** trauma, craniofacial trauma, maxillofacial trauma, prognostic indicators, feline

## Abstract

Craniofacial traumatic injuries contribute significantly to the morbidity and mortality of domestic felines. Previous studies focused on feline craniofacial injuries have investigated the origin of injury, injuries sustained, and effectiveness of diagnostic tools. The aim of the study is to identify prognostic indicators for feline craniofacial trauma patients and determine their association with negative and positive outcomes. The Veterinary Committee on Trauma (VetCOT) Trauma Registry and Dentistry and Oral Surgery Case Logs were utilized to identify feline craniofacial trauma cases that were presented to Colorado State University’s Veterinary Teaching Hospital between 2014 and 2020. Prognostic indicators evaluated included: etiology of injury, signalment (age and sex), the Modified Glascow Coma Scale (MGCS), Animal Trauma Triage (ATT) scores, craniofacial examination findings, diagnostic imaging technique, and injuries identified *via* imaging. Outcomes were determined *via* patient status upon discharge. Outcomes were grouped into the following categories: survival to discharge at initial presentation to CSU Urgent Care (SDIP), survival to discharge after injury treatment/repair by CSU DOSS or another specialty service (SDTX), euthanized due to grave prognosis at initial presentation (EUGP), euthanized due to financial limitations at initial presentation (EUF), and euthanized due to grave prognosis and financial limitations (EUGP + EUF). The continuous data was described using means and standard deviations. To determine the associations of various groupings of clinical signs and imaging findings with outcome a principal component analysis was performed. Patient sex, trauma etiology, cumulative MGCS and ATT scores on initial presentation and clinical signs on initial presentation were identified as prognostic indicators with intact males, vehicular and animal altercations, lower MGCS cumulative scores, higher ATT scores and the presence of altered mentation identified as negative prognostic indicators. Prognostic indicators for feline craniofacial trauma can be associated with outcomes and help guide clinical decision making.

## Introduction

Craniofacial trauma leads to significant morbidity and mortality for feline patients. The head is the second most common region affected in feline trauma cases, and mandibular fractures account for 11.3–23% of all fractures in cats (1, 2). Cats suffering from trauma are almost twice as likely to present for maxillomandibular injuries than non-maxillomandibular lesions (3) Previous studies pertaining to feline craniofacial trauma have focused on trauma etiology, types of injuries sustained, and fracture identification with less emphasis on prognostic indicators and clinical outcomes. The association of outcome with a single etiology, i.e., high rise syndrome, has been previously evaluated with an overall mortality rate of 6% (3), similar to the 8% mortality rate of cats that presented for CT evaluation of skull fractures (4). Overall survival rates of cats that sustain trauma have been reported at 17.5%, and clinical experience indicates that overall assessment of feline craniofacial trauma may indicate even higher mortality rates (5).

Previous craniofacial and feline trauma studies have identified the most common craniofacial fracture locations, number of fractures encountered, incidence of dentoalveolar fractures, and compared the diagnostic accuracy of dental radiography vs. computed tomography for diagnosing craniofacial fractures in canine and feline patients. However, determination of prognostic indicators for feline craniofacial trauma to better guide clinical decision making has not yet been determined (6–14). The goal for this study was to identify prognostic indicators that can be used to determine outcomes in feline patients with craniofacial traumatic injury. A secondary goal was to evaluate the prognosis for various types of maxillofacial injuries determined *via* dental radiographs and computed tomography.

## Materials and methods

Feline head trauma cases that presented to Colorado State University’s Veterinary Teaching Hospital over a 7 year period between 2014 and 2020 were identified by utilizing the Colorado State University VetCOT Trauma Registry and Dentistry and Oral Surgery Service (DOSS) Case Logs. Exclusion criteria included cats dead on arrival, and/or cats with no signs of obvious craniofacial trauma documented in the medical record or animals with exclusively ocular trauma. Prognostic factors identified included etiology of trauma, signalment (age and sex), the Modified Glascow Coma Scale (MGCS; motor activity, brainstem reflexes, level of consciousness) and Animal Trauma Triage (ATT) cumulative scores (perfusion, cardiac, respiratory, eye/muscle/integument, skeletal, and neurological systems), and signs of obvious craniofacial trauma on initial presentation (15). The medical records were reviewed for each patient and were included in the study if sufficient physical examination and historical findings were documented to identify the prognostic indicators described above.

Etiology of trauma was categorized as vehicular trauma, animal altercation (dog or cat bite), human altercation (purposeful or accidental), high rise syndrome, or idiopathic. Signs of obvious craniofacial trauma on initial presentation were recorded as ocular changes, cranial nerve deficits, mandibular fractures, maxillary fractures, TMJ luxation, traumatic induced malocclusion of teeth, dentoalveolar injury, symphyseal separation, epistaxis, inability to close mouth, soft tissue wounds, and altered mentation. Cases that had diagnostic imaging performed, including skull radiographs, dental radiographs and computed tomography, were recorded. The maxillofacial injury diagnoses made *via* imaging were grouped into the following categories: dentoalveolar fractures/injuries, caudal maxillary/zygomatic fractures (maxillary fourth premolar to zygomatic arch), rostral maxillary fractures (canines to maxillary third premolar), incisive/nasal bone fractures, temporomandibular joint luxation, temporomandibular joint fractures, symphyseal separations, mandibular ramus fractures (coronoid and masseteric fossa), mandibular body fractures (mandibular third premolar to first molar), and rostral mandibular fractures (mandibular incisors to canines).

Outcomes were determined *via* patient status upon discharge. Outcomes were grouped into the following categories: survival to discharge at initial presentation to Urgent Care (SDIP), survival to discharge after injury treatment/repair by DOSS or another specialty service (SDTX), euthanized due to grave prognosis at initial presentation (EUGP), euthanized due to financial limitations at initial presentation (EUF), and euthanized due to grave prognosis and financial limitations (EUGP + EUF). To determine associations of each prognostic indicator with outcome the categorical data was described using frequencies and analyzed with a logistic regression model to evaluate the likelihood of an event happening. In order to determine statistical significance for these analyses, outcomes SDIP and SDTX were grouped together as the survival outcome, and outcomes EUGP, EUF, EUGP + EUF were grouped together into the euthanized outcome. The continuous data was described using means and standard deviations. Continuous data was evaluated for normality assumption and analyzed using a Wilcoxon 2-sample test if the data did not meet normality assumption. A value of p of 0.05 was set to determine statistical significance. To determine the associations of various groupings of clinical signs and imaging findings with outcome, a principal component analysis was performed. Principal components were considered significant for eigenvalues of 1.0 or greater. The principal components with the greatest eigenvalues were plotted in a component pattern graph to visualize the associations of clinical signs and imaging findings with outcomes SDIP, SDTX, EUGP, EUGP + EUF.

## Results

One hundred and thirty cases were identified, and 16 cases were excluded resulting in 114 cases meeting the inclusion and exclusion criteria. Eighteen cases were identified solely from the DOSS Case Logs, 76 cases were identified exclusively from the VetCot Trauma Registry, and 20 cases were identified from the CSU DOSS Case Logs and the VetCot Trauma Registry. The overall survival rate when combining outcomes SDIP and SDTX was 62.3%. The overall euthanasia rate including the 3 cases that died while in the Urgent Care was 37.7% (see Table 1). The percentage of cats that received surgery and survived to discharge was 47.4% (54/114). After initial treatment and evaluation in the Urgent Care, 16.7% (19/114) cases survived to discharge with 2 out of those 19 cases either being transferred to the Larimer Humane Society with a grave prognosis or discharged to the primary care veterinarian for euthanasia.

**Table 1:** Number of cases associated with outcomes.

Outcome	Number of cases
Survival to discharge at initial presentation (SDIP)	19*
Survival to discharge after injuring treatment/repair (SDTX)	54
Euthanized due to grave prognosis (EUGP)	21
Euthanized due to financial limitations (EUF)	2
Euthanized due to grave prognosis + financial limitations (EUGP + EUF)	15
Died	3
Total cases	114

* 2 of the 19 cases that survived to discharge at initial presentation to Urgent Care were transferred to the care of primary care veterinarians for euthanasia due to grave prognosis.

### Signalment

**Figure 1:**
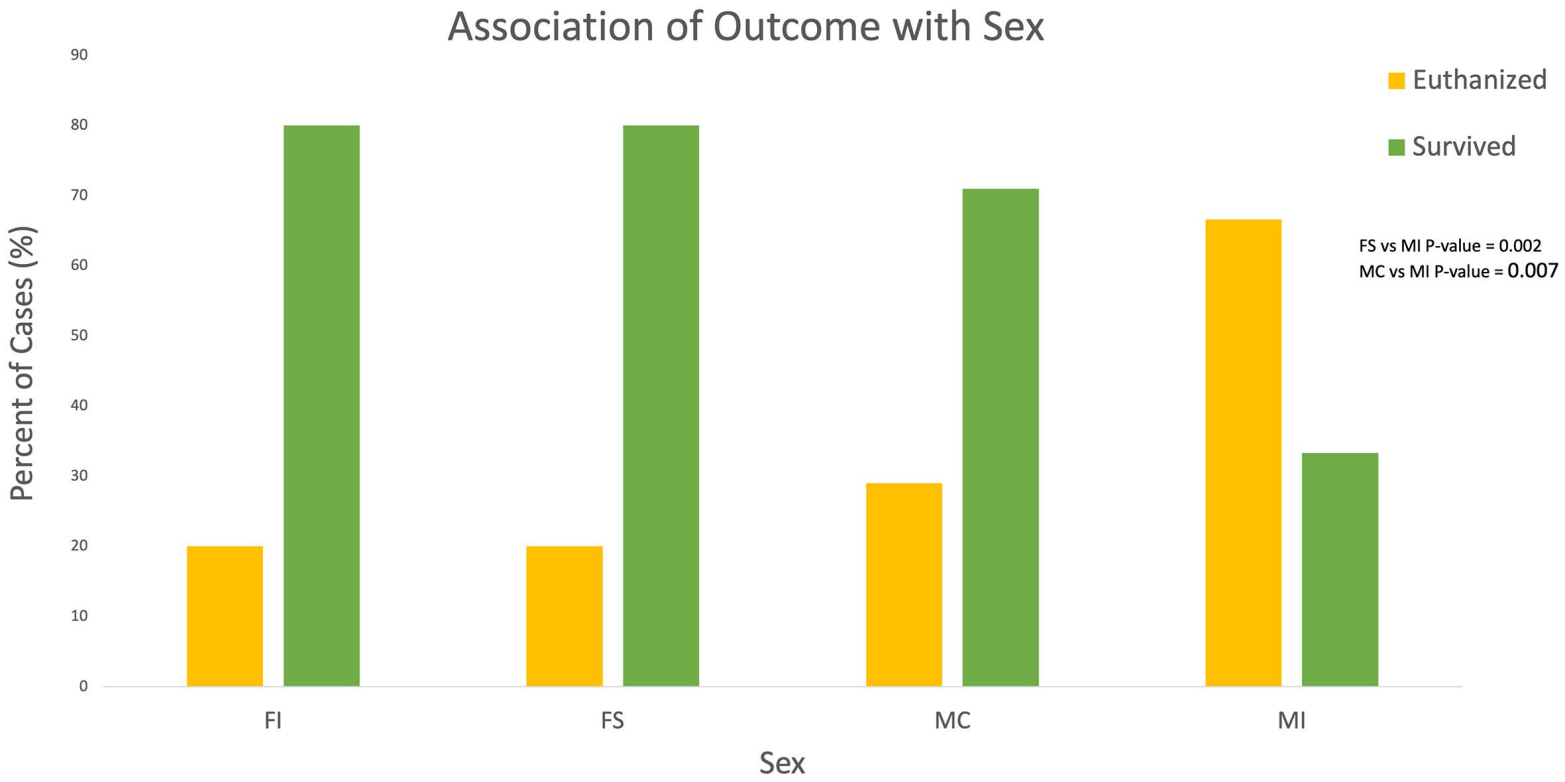
Associations of outcome with sex.

The age range of the analyzed cases was 1–232 months with a median of 42 months and mean of 63.4 months. Age was determined to be not statistically associated with outcome when the outcomes were grouped into survived vs. euthanized with a *p*-value of 0.46. For population sex, 47.4% (54/114) of patients were male castrated (MC), 15.7% (18/114) were male intact (MI), 27.2% (31/114) were female spayed (FS), 5.3% (6/114) cases were female intact (FI), and 4.4% (5/114) were of unknown sex (U). Cats with unknown sex were excluded from statistical analysis. Eight (8/18) 44.4% male intact individuals were euthanized due to grave prognosis vs. 6/54 11.1% male castrated and 4/31 12.9% female spayed individuals, respectively. Sex was statistically significantly associated with outcome when outcome was grouped into either euthanized or survived. The probability of euthanasia was significantly lower for FS individuals when compared to MI (*p*-value 0.002), and the probability of euthanasia was significantly lower in MC individuals when compared to MI (*p*-value 0.007; see Figure 1).

### Trauma etiology and type

**Figure 2:**
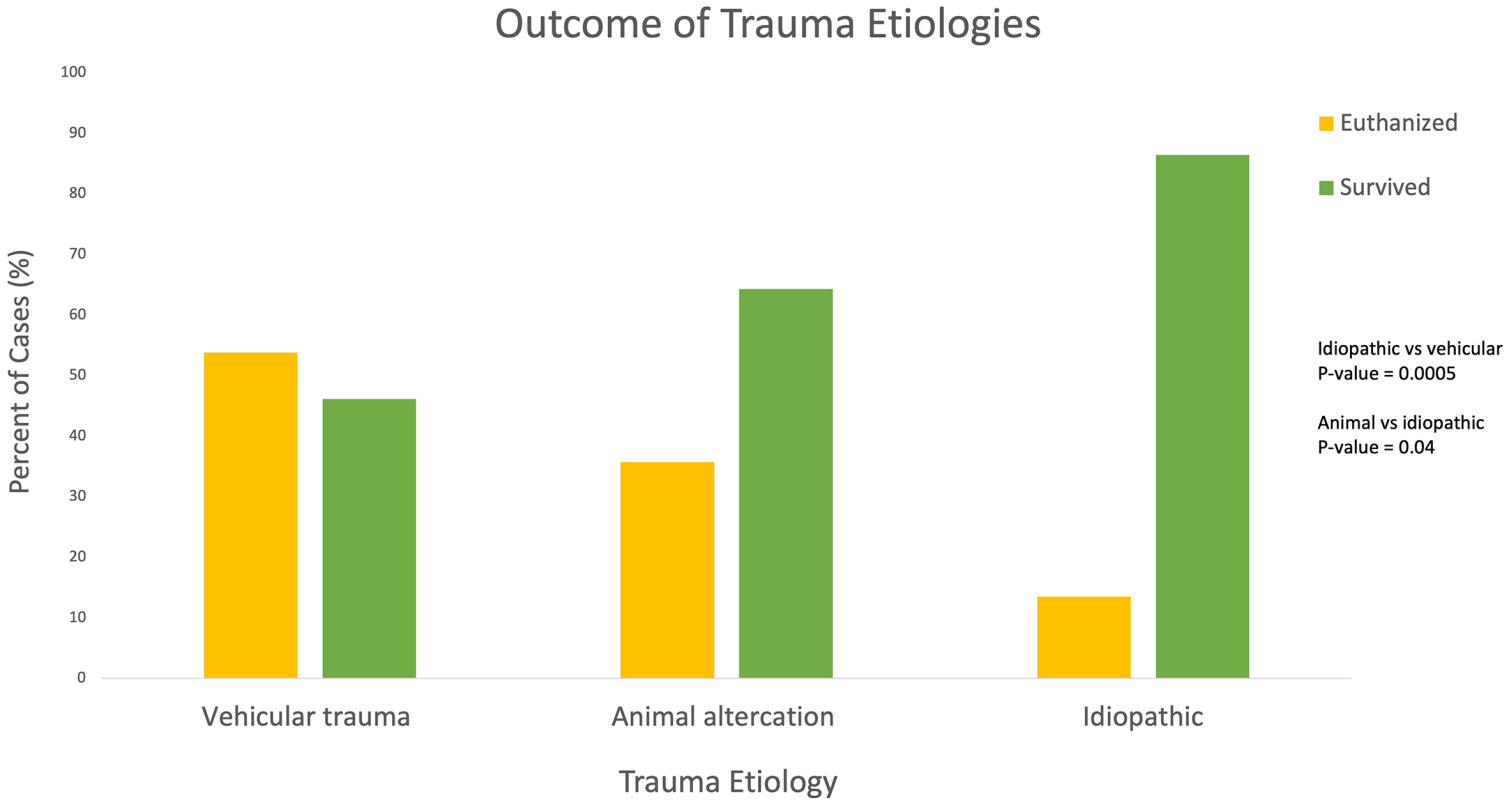
Outcome of trauma etiologies.

Four cases were excluded from statistical analysis due to their etiologies not fitting into the defined etiology categories (1 jump off of chair, 1 ran into wall, 1 self-traumatization, 1 caught in recliner) resulting in a total of 110 etiologies for analysis. Vehicular trauma was the most common etiology with 39/110 (39.5%) cases, followed by idiopathic/unknown 37/110 (33.6%), and animal altercation 30/110 (27.3%). The remainder of the cases were caused by human altercation 3/110 (2.7%), and high-rise syndrome 1/110 (0.9%). Trauma etiology was found to be statistically significantly associated with outcome. The probability of euthanasia is significantly lower when the trauma etiology is idiopathic as compared to vehicular trauma (*p*-value 0.0005). The probability of euthanasia is significantly higher when the trauma etiology is animal altercation as compared to idiopathic (*p*-value 0.04; see Figure 2). Therefore, patients with trauma etiologies of vehicular trauma or animal altercations were significantly associated with negative outcomes.

### Modified Glascow coma scale (MGCS) and animal trauma triage score (ATT)

**Figure 3:**
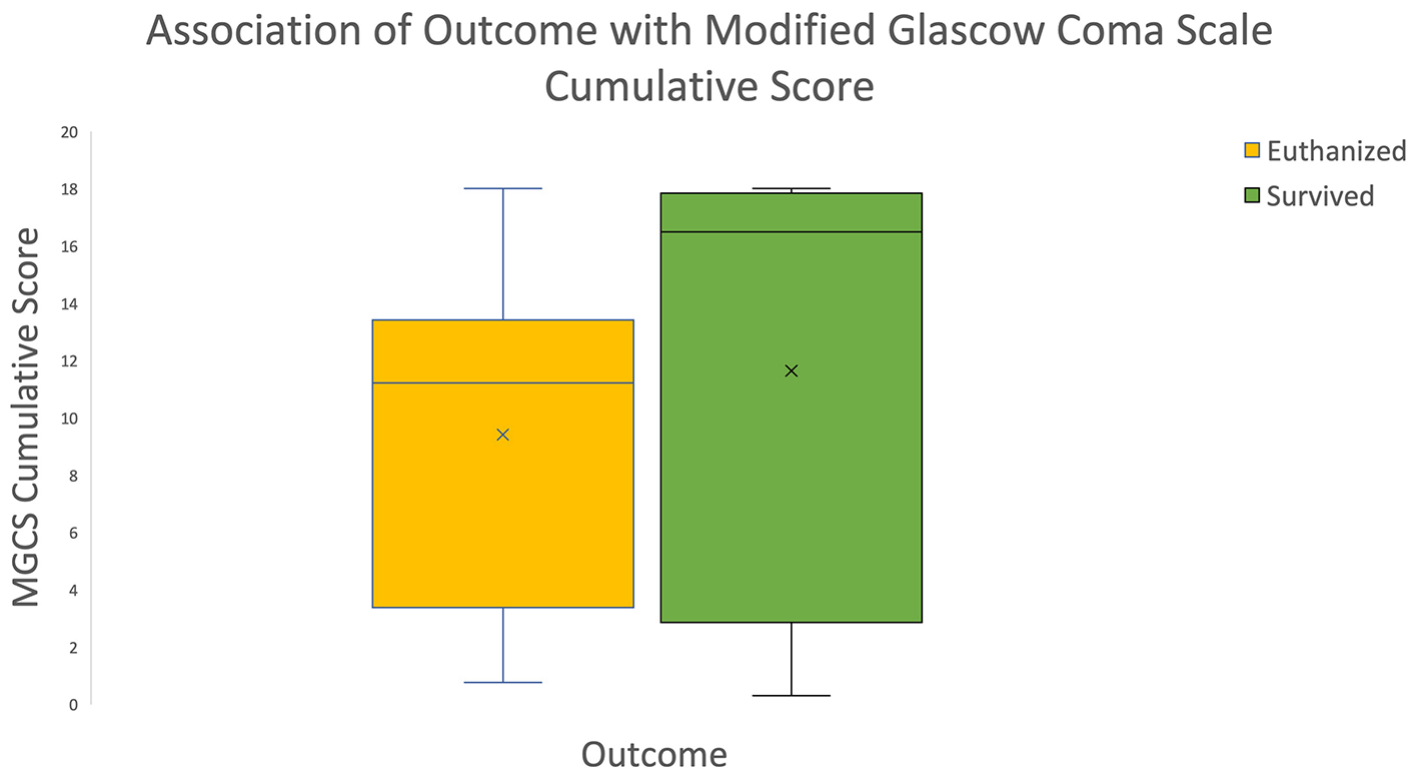
Association of outcome with Modified Glascow Coma Scale Cumulative Scores The box and whisker plot demonstrates that the median MGCS cumulative score was significantly lower in euthanized animals compared to the ones that survived to discharge. The lower the MGCS cumulative score the graver the prognosis.

**Figure 4:**
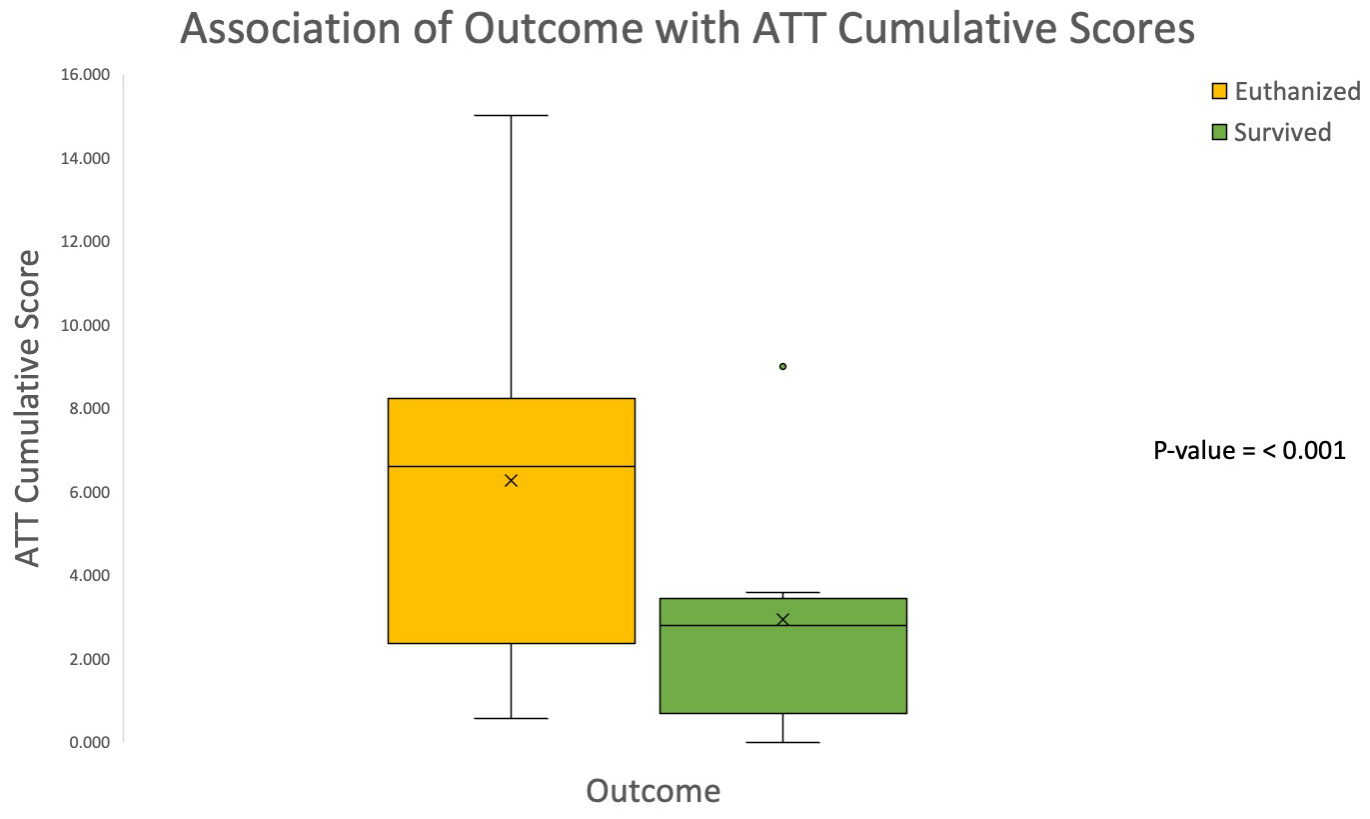
Association of outcome with Animal Trauma Triage (ATT) cumulative scores The box and whisker plot demonstrates that the Animal Trauma Triage cumulative score was significantly higher in patients that were euthanized. The higher the ATT cumulative score the graver the prognosis.

The cumulative MGCS and ATT scores were obtained for each patient. The outcomes were grouped into survived or euthanized for analysis purposes. The median cumulative MGCS scores were significantly lower in euthanized animals compared to those that survived to discharge (*p*-value < 0.001; see Figure 3). These findings are consistent with the previously validated MGCS in which lower scores are associated with a more grave prognosis. The median cumulative ATT scores were significantly higher in euthanized animals compared to those that survived to discharge (*p*-value < 0.001; see Figure 4). These results are also consistent with the previously validated ATT scoring system in which higher scores are associated with a more grave prognosis.

### Signs of obvious craniofacial trauma

The presence of obvious signs of craniofacial trauma on initial presentation were identified in the patient’s medical record and recorded. Sixty six percent of the cases in this study were solely examined by Urgent/Critical Care interns, residents and faculty vs. Dentistry and Oral Surgery residents and faculty. Soft tissue wounds, ocular changes and epistaxis were the most common signs of craniofacial trauma noted, followed by altered mentation and obvious mandibular fractures (see Table 2). Dentoalveolar injuries were only identified in 13 cases on initial presentation to the Urgent Care. The probability of euthanasia was significantly lower when soft tissue wounds were present (*p*-value 0.02). The probability of euthanasia was significantly higher when altered mentation was present on initial presentation (p-value < 0.001). Maxillary and mandibular fractures were not statistically significantly associated with outcome (*p*-values 0.1, 0.19). There were a total of 12 clinical signs reported either alone or in a combination among each of the cases. Statistical analyses were not performed to find any associations of each of those signs with trauma conditions. Instead, a principal component analysis was performed to determine the association of various clinical signs with each other and outcome (see “Principal component analysis section” below).

**Table 2:** Number of cases that demonstrated obvious signs of craniofacial trauma.

Obvious signs of craniofacial trauma	Number of cases
Soft tissue wounds	57
Ocular changes	48
Epistaxis	37
Altered mentation	34
Mandibular fractures	31
Symphyseal separations	20
Inability to close mouth	19
Cranial nerve deficits	15
Maxillary fractures	15
Dentoalveolar injury	13
Facial swelling	12
TMJ luxations	8
Traumatic induced malocclusion of teeth	8

### Craniofacial injuries identified on diagnostic imaging

Forty-six (46/114) cases had diagnostic imaging performed with 57 total imaging modalities for all cases. Dental radiographs were the most common imaging modality (28/57), followed by CT (19/57), and skull radiographs (10/57). Skull radiographs were never the sole imaging modality performed for a case. One hundred and nine total injuries were diagnosed *via* imaging, and the most common injuries included symphyseal separation (23/109), dentoalveolar fracture (21/109), mandibular ramus fracture (17/109), and mandibular body fracture (13/109). see Table 3. Single imaging pathologic findings were only present for a total of 6 cases which included 3 dentoalveolar fractures, 2 TMJ luxations, and 1 singular mandibular ramus fracture. There were a total of 10 imaging findings reported either alone or in a combination among each of the cases. Statistical analyses were not performed to find any associations of each of those signs with trauma conditions. Instead, a principal component analysis was performed to determine the association of various imaging findings with each other and outcome (see “Principal component analysis section” below).

**Table 3:** Number and type of injuries identified on diagnostic imaging.

Injuries identified on imaging	Number of injuries identified on imaging
Symphyseal separation	23
Dentoalveolar fractures	21
Mandibular ramus fractures	17
Mandibular body fractures	13
TMJ luxations	9
Caudal maxillary/zygomatic fractures	8
Incisive/nasal bone fractures	6
Rostral maxillary fractures	5
TMJ fractures	4
Rostral mandibular fractures	3
Total	109

### Principal component analysis: associations of obvious signs of craniofacial trauma with outcome

**Figure 5:**
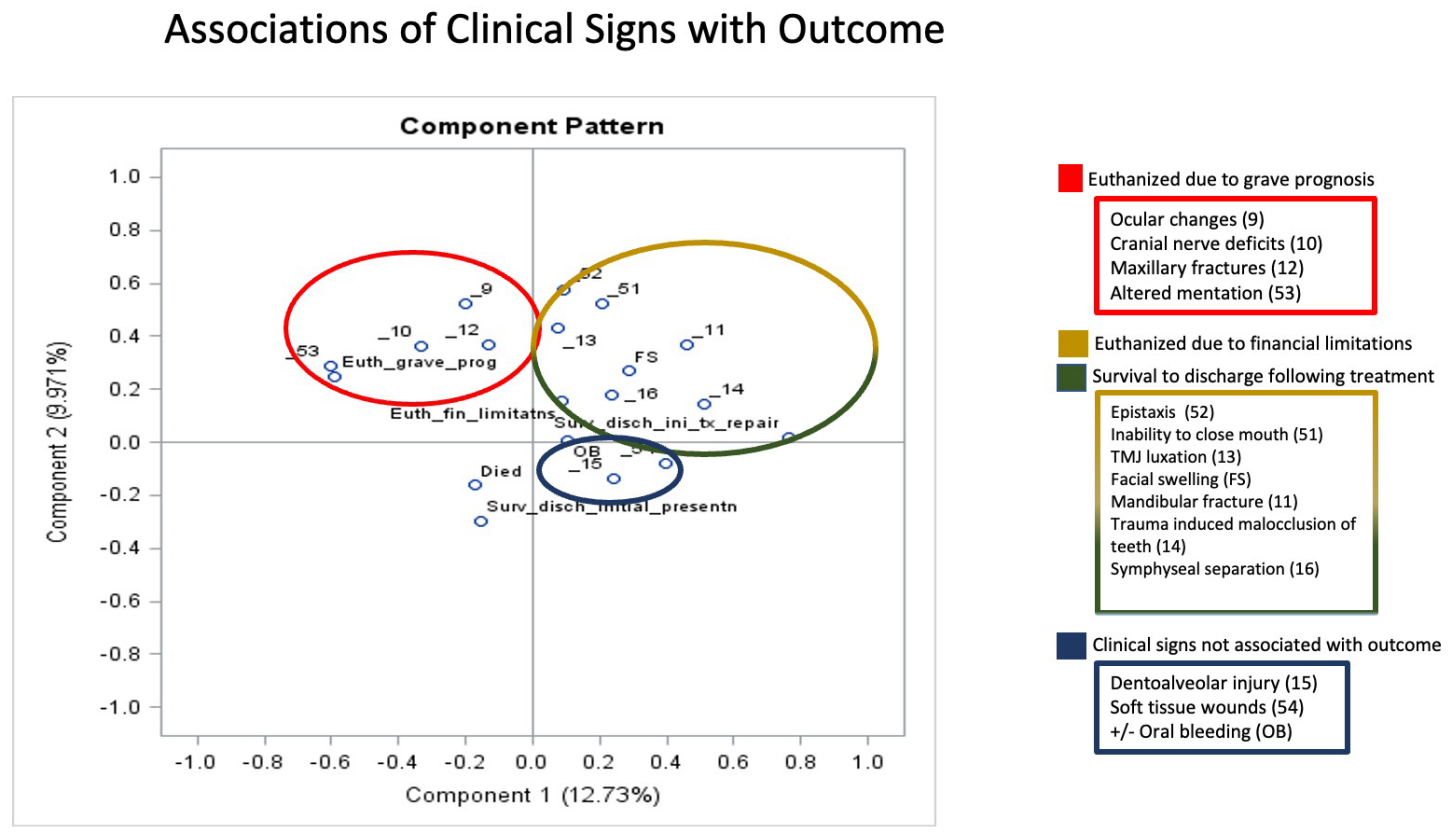
Principal Component Analysis Associations of Clinical Signs with Outcome Components 1 and 2 had the greatest eigenvalues and were plotted in a component pattern graph to visualize the associations of clinical signs with outcome. The clinical signs associated with euthanized due to grave prognosis (EUGP) are circled in red in the upper left quadrant. The clinical signs associated with euthanized due to financial limitations (EUF) and survival to discharge following treatment (SDTX) are circled in yellow and green, respectively, in the upper right quadrant. The clinical signs associated with each other but no outcome are circled in blue in the lower right quadrant. The outcomes of died and survival to discharge at initial presentation (SDIP) were not associated with any clinical signs in the lower left quadrant.

**Figure 6:**
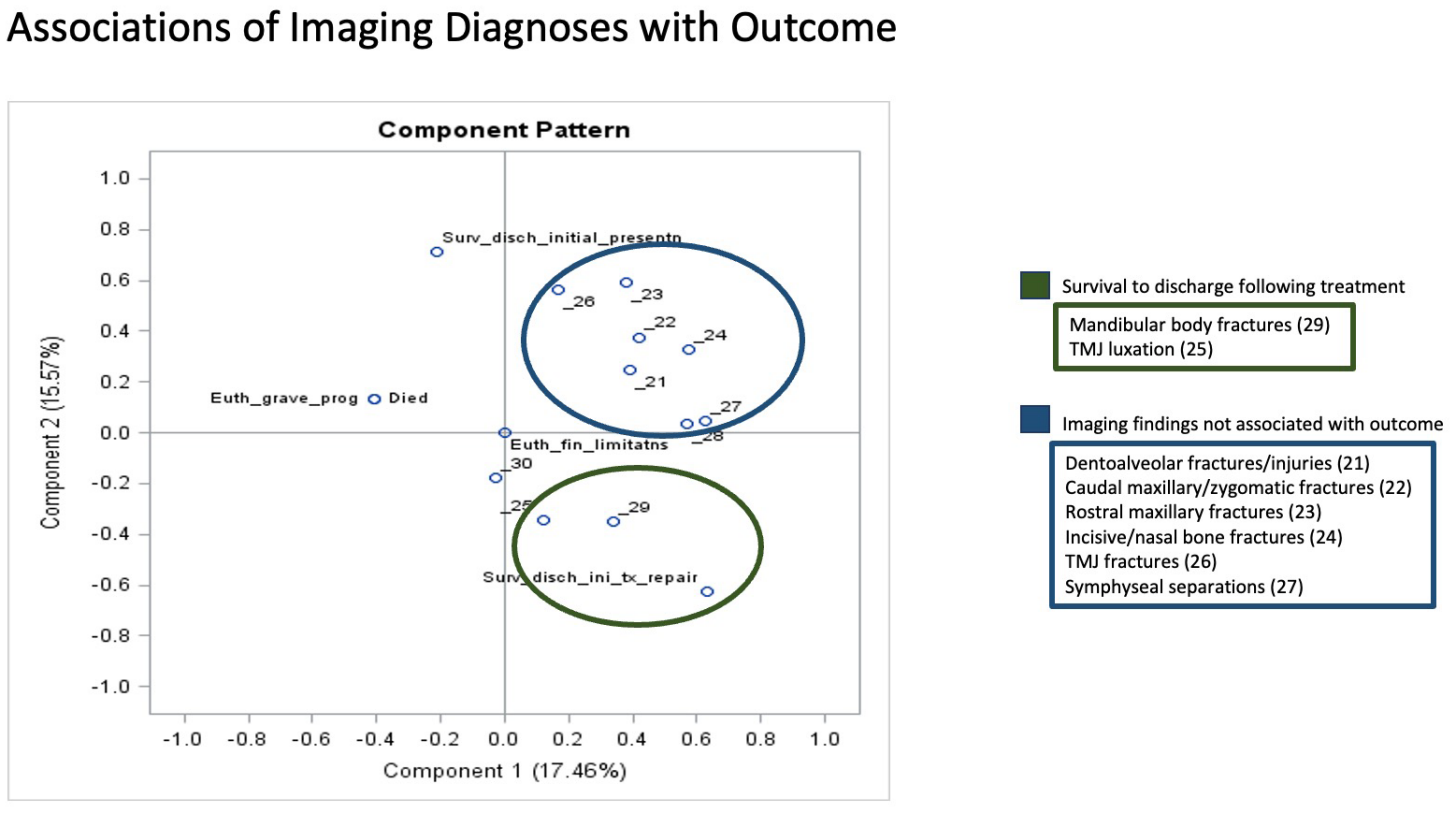
Principal Component Analysis Associations of Imaging Diagnoses with Outcome Components 1 and 2 had the greatest eigenvalues and were plotted in a component pattern graph to visualize the associations of imaging diagnoses with outcome. The imaging findings associated with survival to discharge following treatment (SDTX) are circled in green in the right lower quadrant. The imaging findings not associated with outcome are circled in blue in the right upper quadrant. The outcomes survival to discharge at initial presentation (SDIP), euthanized due to grave prognosis (EUGP) and the patients that died at initial presentation were not associated with any imaging diagnoses.

To determine the associations of various groupings of clinical signs with outcomes SDIP, SDTX, EUGP, EUF, EUGP + EUF, and died, a principal component analysis was performed. Component pattern plots were utilized to determine associations between variables. When components 1 and 2 were plotted associations between outcome EUGP were found with the clinical signs ocular changes, cranial nerve deficits, maxillary fractures and altered mentation (see Figure 5). Outcomes SDTX and EUF were associated with the clinical signs epistaxis, inability to close mouth, TMJ luxations, facial swelling, mandibular fractures, trauma induced malocclusion of teeth, and symphyseal separations (see Figure 6). The clinical signs dentoalveolar injury, soft tissue wounds, +/− oral bleeding due its proximity to the *x*-axis, were not associated with any outcomes according to the component pattern plot.

### Principal component analysis: associations of diagnostic imaging findings with outcome

When components 1 and 2 were plotted on a component pattern plot, the only imaging findings associated with an outcome, outcome SDTX, were mandibular body fractures and TMJ luxations. Outcome EUGP and died were closely connected but not associated with any imaging findings. Dentoalveolar fractures/injuries, caudal maxillary/zygomatic fractures, rostral maxillary fractures, incisive/nasal bone fractures, TMJ fractures and symphyseal separations were associated together but not with any outcomes.

## Discussion

The survival rate in this study was 62.3%, which is lower than previously reported for overall feline traumatic survival rates of 82.5 and 82% (16, 17). The discrepancy between cats that presented to Urgent Care facilities for traumatic injury to all areas of the body versus craniofacial trauma indicates that cats who experience craniofacial trauma have a higher risk of poor outcome. In this study, 47.4% of cats received definitive surgical treatment and survived to discharge. A 2018 study by Hall et al. found that out of 3,425 cats that presented to VetCOT identified veterinary trauma centers for traumatic injuries, 36.2% of cats received surgery (6). The difference between the percentage of cats that received surgical treatment for general trauma and craniofacial trauma may indicate that craniofacial trauma often necessitates the need for surgery.

The mortality rate of 37.7% in the present study differs from other studies that evaluate the mortality rates of feline craniofacial trauma patients. One study evaluating cats that sustained skull fractures diagnosed on CT found an 8% mortality rate with no risk factors for mortality identified (5). Another study focusing on mortality of cats with high rise syndrome found an overall mortality rate of 6% (3). The vast difference between the mortality rates identified in the present study versus previous studies was likely due to the inclusion of all forms of craniofacial trauma cases. One out of 19 (5.2%) cases receiving CT imaging in the present study was discharged to the primary care veterinarian for euthanasia due to identification of a concurrent brain tumor and severe craniofacial injuries. These findings indicate that survival to CT imaging may be a positive prognostic indicator given the significantly higher survival rates of patients that received CT imaging. High rise syndrome was the etiology for only 1 case in the present study due to the relative lack of high rise structures in Northern Colorado, and it was excluded from Wilcoxon 2-sample test statistical analysis. It is possible that if greater percentage of trauma etiologies had been high rise syndrome the overall survival rate would have been altered. The etiologies of animal altercation, vehicular trauma and idiopathic trauma were statistically significantly associated with outcome with idiopathic etiology serving as a positive prognostic indicator and animal altercation and vehicular trauma serving as negative prognostic indicators.

When evaluating patient signalment as a potential prognostic indicator, the results of the present study found that age was not significantly associated with outcome. Other studies have demonstrated an association between young animals being more likely to experience traumatic events, but the association of feline craniofacial trauma patient age with outcome has not previously been documented to the author’s knowledge (2, 18, 19). The median and mean age of patients in the present study was 42 and 63.4 months which is higher than previously reported studies (2, 20). A risk factor for the development of complications in feline skull fracture cases has been previously identified as increasing age, but a statistically significant association of age with outcome has not been documented (4). These findings indicate that although younger patients may be overrepresented in maxillofacial trauma cases, the patient’s age is not an effective prognostic indicator for outcome. The results of this study found that patient sex was statistically significantly associated with outcome with male intact individuals being more likely to be euthanized as compared to male castrated and female spayed individuals. Eight (8/18) 44.4% male intact individuals were euthanized due to grave prognosis vs. 6/54 11.1% male castrated and 4/31 12.9% female spayed individuals, respectively. Although it has been previously speculated that intact male individuals are more likely to wander, get in fights and sustain trauma, no previous studies have reported an association between sex and outcome in feline craniofacial trauma cases. The results of the present study indicate that male intact individuals are more likely to sustain severe injuries resulting in a grave prognosis. Breed differences were not evaluated in this study due to the high proportion of domestic shorthair cats in the study population, and due to the fact that domestic shorthair, long and medium haired cats (the most prevalent breeds) do not have significant size variations as do different dog breeds.

The previously validated MGCS and ATT cumulative scores were significantly associated with outcome in the present study. Patients with lower MGCS scores on initial presentation were significantly more likely to be euthanized, and patients with higher ATT scores were significantly more likely to be euthanized. These results were consistent with these previously validated scales and indicate that lower MCGS scores and higher ATT scores on initial presentation are negative prognostic indicators for survival of feline craniofacial trauma patients. The association of altered mentation on initial presentation with euthanasia is supported by the MGCS and ATT score results. Only 32.3% of cases that had altered mentation on initial presentation survived to discharge. These results are consistent with the MGCS and ATT scores in which patients with more severely altered mentation have worse prognoses. The present study did not separately assess the association of various altered mentation states with outcome, but instead used the cumulative MGCS and ATT scores to reflect increases in mentation/central nervous system compromise. The results indicate that the presence of altered mentation on initial presentation for feline craniofacial trauma was a negative prognostic indicator.

When individual obvious signs of craniofacial trauma on initial presentation were associated with outcome, soft tissue wounds were significantly associated with survival to discharge, altered mentation was significantly associated with euthanasia and mandibular and maxillary fractures were not associated outcome. These findings may be influenced by the fact that there were more soft tissue wounds and altered mentation identified than signs of maxillary and mandibular fractures on initial presentation. The differences in association with outcome may be due to the fact that maxillary and mandibular fractures are not always evident on physical exam.

Sixty six percent of the cases in this study were solely examined by Urgent/Critical Care interns, residents and faculty not Dentistry and Oral Surgery residents and faculty. In a study that evaluated the differences in detection of orofacial manifestations of high rise syndrome in cats between Emergency/Critical Care veterinarians and Dentistry and Oral Surgery residents and faculty, orofacial findings were identified in 100% of cases examined by Dentistry and Oral surgery residents and faculty versus 59.4% of cases examined by Emergency/Critical Care residents and faculty (3). The difference in identification and recording of injuries between the two groups may have likewise skewed the results of the present study. In Bonner’s 2011 paper, dental trauma was identified in 6 vs. 71.4% of the Emergency veterinarians vs. Dentistry and Oral surgery veterinarians respectively, which is consistent with the percentage of cases with dentoalveolar injury identified on initial presentation in the present study (11.4%). It is likely that the incidence of dentoalveolar injury would have been higher if all cases were examined by a Dentistry and Oral Surgery resident or faculty.

The principal component plots revealed that the constellation of clinical signs including ocular changes, cranial nerve deficits, maxillary fractures and altered mentation were associated with the outcome euthanasia due to grave prognosis. This combination of clinical signs is consistent with central nervous system compromise and traumatic brain injury. When these signs are present on initial presentation, they can serve as a negative prognostic indicator. There was overlap of the outcomes survival to discharge following injury treatment/repair and euthanized due to financial limitations for the constellation of signs epistaxis, inability to close mouth, TMJ luxations, facial swelling, mandibular fractures, trauma induced malocclusion of teeth, and symphyseal separations. The association of both outcomes with these clinical signs indicates that finances can be the limiting factor for a successful outcome for these feline craniofacial trauma patients.

To the author’s knowledge no previous studies have focused on identifying prognostic indicators for feline craniofacial trauma. A previous multicenter evaluation of canine trauma patients found that 30 out of 315 dogs did not survive to discharge and 53.3% were euthanized due to financial reasons (17). In this study only 2/114 cases were euthanized solely due to financial reasons with 15/114 cases being euthanized due to the combination of financial reasons and grave prognosis. The difference in reason for euthanasia may be due to this study’s focus on exclusively craniofacial trauma.

The principal component analysis of imaging findings and outcome found that mandibular body fracture and TMJ luxation were associated with survival to discharge following injury treatment/repair. No other imaging findings were associated with outcome *via* the principal component analysis. All patients that had imaging performed (CT, dental radiographs and skull radiographs) survived to discharge following surgical repair of their injuries with the exception of 1 case that had a skull CT performed which identified an intracranial mass. These injuries included dentoalveolar fractures, caudal maxillary/zygomatic fractures, rostral maxillary fractures, TMJ fractures, and symphyseal separation. The 5.2% mortality rate of patients that received CT scans is in stark contrast to the overall mortality rate of 37.7% of all feline craniofacial trauma patients. The cases that had dental radiographs and skull CT scans performed had been deemed stable enough for treatment following their initial triage exam, and the owners had sufficient finances to pursue referral to the Dentistry and Oral Surgery specialty service. This study demonstrates that overall survival rates of craniofacial trauma in cats are likely significantly lower than those demonstrated in previous studies since the majority of the literature available either evaluates overall trauma patients without a focus on the craniofacial region or evaluates patients that survived to treatment to receive diagnostic imaging. In this study, there were more associations of clinical signs of craniofacial trauma on initial presentation with outcome than there were associations of imaging findings with outcome. The authors interpret this difference to indicate that evaluation of prognostic indicators such as patient sex, trauma etiology, MGCS and ATT cumulative scores, and signs of obvious craniofacial trauma on initial presentation can help create a realistic clinical picture and likelihood of survival for cats suffering from craniofacial trauma.

Limitations of this study primarily stem from its retrospective nature. Searching the VetCOT Trauma Registry Database for feline head trauma cases yielded a significant portion of the cases in this study, but there was insufficient detail within the VetCOT database for a retrospective evaluation of craniofacial trauma prognostic indicators. There were also frequent significant discrepancies between the VetCOT data and the medical record. The medical record was utilized as the gold standard, but since the data was collected retrospectively there may have been errors in the record. The diagnostic imaging studies were not directly reviewed by the authors; therefore, errors in diagnoses made by the Dentistry and Oral Surgery residents and faculty in the medical records may have occurred. This study aimed to assess feline trauma patients that presented to CSU’s Urgent Care regardless of if they received advanced diagnostic imaging. Since computed tomography was not performed for each patient, the number and extent of craniofacial injuries was likely underestimated which may have impacted the results of the study. Further prospective studies are needed to more accurately determine the accuracy of the identified prognostic indicators for predicting outcomes for feline craniofacial trauma. This study does not include long-term follow-up after discharge from the hospital. In the authors’ experience, when appropriate clinical and treatment decision making is utilized, maxillofacial trauma patients rarely experience mortality due to catastrophic treatment failure after discharge. Further studies are needed to evaluate long-term survival and morbidity associated with maxillomandibular trauma repair.

## Conclusion

The results of the current study indicate that feline craniofacial trauma patients are at an increased risk for mortality than the general trauma patient population. Prognostic indicators such as patient sex, trauma type and etiology, cumulative MGCS and ATT scores on initial presentation and clinical signs on initial presentation can help provide prognostic information and guide treatment and decision making for veterinary emergency practitioners and surgical specialists alike. Further studies are needed to determine the long-term prognosis following discharge of feline craniofacial trauma patients and the impact of these prognostic indicators on long term prognosis.

## Data availability statement

The original contributions presented in the study are included in the article/Supplementary material. Further inquiries can be directed to the corresponding author.

## Ethics statement

Ethical review and approval was not required for the animal study because this was a retrospective study in which patient medical records were reviewed. No clients were contacted for further information. No prospective date was obtained.

## Author contributions

JK, NH, SR, and JR contributed to the conception, design of the study, and revised the manuscript. JK organized the database and wrote all drafts of the manuscript. SR performed the statistical analysis. All authors contributed to the article and approved the submitted version.

## Funding

This work was supported by Colorado State University Young Investigators Grant Program 2021.

## Conflict of interest

The authors declare that the research was conducted in the absence of any commercial or financial relationships that could be construed as a potential conflict of interest.

## Publisher’s note

All claims expressed in this article are solely those of the authors and do not necessarily represent those of their affiliated organizations, or those of the publisher, the editors and the reviewers. Any product that may be evaluated in this article, or claim that may be made by its manufacturer, is not guaranteed or endorsed by the publisher.

## Abbreviations

DOSS: Dentistry and Oral Surgery Service
MGCS: Modified Glascow Coma Scale
ATT: Animal Trauma Triage
SDIP: Survival to discharge at initial presentation to Urgent Care
SDTX: Survival to discharge after injury treatment/repair by DOSS or another specialty service
EUGP: Euthanized due to grave prognosis at initial presentation
EUF: Euthanized due to financial limitations
EUGP + EUF: Euthanized due to grave prognosis and financial limitations.
